# The Effectiveness of First-Generation iStent Microbypass Implantation Depends on Initial Intraocular Pressure: 24-Month Follow-Up—Prospective Clinical Trial

**DOI:** 10.1155/2020/8164703

**Published:** 2020-06-23

**Authors:** Joanna Konopińska, Milena Kozera, Paweł Kraśnicki, Zofia Mariak, Marek Rękas

**Affiliations:** ^1^Department of Ophthalmology, Medical University of Białystok, M. Sklodowskiej-Curie 24A STR, 15-276 Białystok, Poland; ^2^Department of Ophthalmology, Military Institute of Medicine, Szaserów 128 STR, 04-141 Warsaw, Poland

## Abstract

**Background:**

Evaluation of efficacy of the iStent trabecular bypass implant in reducing intraocular pressure (IOP) depending on the value pretreatment IOP and number of medications used before surgery in patients with primary open-angle glaucoma (POAG) and pseudoexfoliative glaucoma (PXG) and coexisting cataract.

**Methods:**

A prospective, uncontrolled, interventional case series. 72 patients, on a mean age of 72.42 ± 9.17, were divided into two groups depending on baseline IOP: group I < 26 mmHg and group II ≥ 26 mmHg. All subjects underwent *ab interno* implantation of a single iStent together with cataract surgery. Best-corrected visual acuity (BCVA), IOP, number of antiglaucoma medications, visual field, and number and type of complications were examined before and after surgery. Postoperative patients were followed up at 1, 7, and 30 days and 3, 6, 12, and 24 months. All the patients were washed out preoperatively as well as postoperatively.

**Results:**

The mean observation time was 20 months. The mean preoperative IOP was 21.03 ± 1.44 mmHg in group I and reduced to mean 15.60 ± 2.12 mmHg after operation. In group II, mean IOP reduced from 26.00 ± 0.00 to 18.56 ± 1.81 (*p*=0.003). Mean glaucoma medications decreased from 1.35 ± 0.65 to 0.29 ± 0.52 in group I (*p* < 0.001) and from 2.89 ± 1.18 to 1.33 ± 1.50 in group II (*p* < 0.001). At 24 months, medication reduction was significantly greater in group I than group II (*p*=0.026).

**Conclusions:**

Combined cataract surgery with implantation of iStent seems to be an effective procedure in patients with mild-to-moderate open-angle glaucoma and cataract. In patients with baseline IOP < 26 mmHg, surgery reduced IOP and medication use significantly declined to 2 years, with greater reductions achieved versus patients with baseline IOP ≥ 26 mmHg. This trial is registered with NCT03807869.

## 1. Introduction

The search for effective and safe surgical procedures in the treatment of glaucoma has been ongoing since the introduction of the first filtration operations in 1910 [1]. Trabeculectomy has been the gold standard for antiglaucoma treatments for many years. It offers control over IOP, but at the same time it carries a great risk of postoperative complications including hypotony, hemorrhagic choroidal detachment, or flattening of the anterior chamber of the eye [2]. Additionally, fibrosis of the scleral flap lowers the rate of success of filtration procedures over time [3]. These prompted researchers to search for safer operational techniques while maintaining their high efficiency.

Intensive research on low-invasive glaucoma surgery and its application have been conducted for over 10 years. Those procedures are known as microinvasive glaucoma surgery (MIGS) such as iStent developed by Glaukos (Glaukos Corporation, Laguna Hills, CA, USA). iStent consists of single microtube made of titanium covered with heparin and it is placed directly into Schlemm's canal *ab interno* with the use of spring mechanism.

To date, a substantial amount of randomized controlled trials has shown mixed effectiveness of procedures using iStent implants. iStent is indicated to patients with mild-to-moderate glaucoma and visually significant cataract, stable under topical medical therapy with the aim of lowering the medication burden. Postsurgical decrease of IOP resulted in 9% to 33% [4–8], and the amount of medication declined to 0.5–2.0 post op [5, 8–10]. This rather big difference in the effectiveness of the implant procedure gave basis to suggest that there may be a way of achieving a higher success rate.

In healthy eye, the aqueous humor flows via ciliary body and trabecular meshwork into the collector channels that carry it to the episcleral veins [11]. In POAG, juxtacanalicular meshwork offers greater resistance to the outflow of the aqueous humor, leading to an increase in IOP. This mechanism is not fully understood [12, 13]. MIGS procedures targeting trabecular meshwork work by creating a bypass between the anterior chamber and collector channels. Battista et al. used bovine eyes to study the effects of increased IOP in the range of 7 to 45 mmHg on Schlemm's canal [14]. They showed that increasing IOP coincides with twofold reduction of effective aqueous humor outflow [14]. Also increasing IOP to 45 mmHg causes a progressive collapse of Schlemm's canal and its walls herniate into the outlet of the water collectors leading to further outflow obstruction. They also found that 95% of collectors were blocked when IOP < 30 mmHg.

In another study, Grieshaber showed, with IOP higher than 20 mmHg, that the number of closed collectors increases, and at IOP exceeding 25–30 mm Hg, most of collector channels are closed and Schlemm's canal collapses [15]. From this experiment, the conclusion is that improving the outflow via trabecular meshwork at IOPs above 25 mmHg is unlikely to bring the expected drop in IOP. The effectiveness of iStent depends on the permeability of the posttrabecular system, which is still obstructed; therefore, the efficacy of the iStent microbypass will be limited.

The aim of our study was to assess the effectiveness of iStent implant procedures based on preoperative IOP values as well as the number of medications used.

## 2. Materials and Methods

The study protocol was in line with the principles of the Declaration of Helsinki and was designed according to the standards of Good Clinical Practice and approved by the Bioethics Committee at the Military Medical Institute in Warsaw.

The criteria for inclusion required glaucoma and concomitant cataracts (NC1, NC2) according to LOCS III. Patients with POAG and PXG in whom the target IOP level was not achieved, despite the maximally tolerated both local and general antihypertensive treatment, were also qualified for the procedure. Patients enrolled to the study had discontinued medications from the eye qualified for the procedure. In the case of prostaglandins and beta-blockers, this period lasted 4 weeks, and with alpha-adrenergic drugs and carbonic anhydrase inhibitors, it lasted 2 weeks. The final inclusion criteria were evaluated in comparison to the baseline visit. It was an IOP level between 18 and 36 mmHg, visual acuity no worse than 0.1 (according to Snellen's notification), and a clearly visible sclera in the gonioscopic examination.

Additional inclusion criteria were as follows: documented progression of visual field defects, significant daily IOP fluctuations, lack of patient cooperation in the use of antiglaucoma therapy, and allergy to topical drugs. Legally binding, conscious informed consent was obtained from every patient prior to their recruitment in the trial. All patients agreed to participate in the study for a period of at least 24 months after informing them about the nature of the procedure and other surgical alternatives.

The exclusion criterion was the lack of consent to participate in the study, previous surgical and laser procedures in the eye, narrow or primary angle-closure glaucoma (PACG), postinflammatory or posttraumatic glaucoma, chronic corneal disease, and corneal opacity that prevent gonioscopic assessment, optic nerve disease, advanced disease macular degenerative, active inflammatory process, pregnancy, and general steroid therapy. All the patients have been washed out preoperatively as well as postoperatively as described further.

The prospective study included 72 eyes in which combined antiglaucoma procedures were performed: phacoemulsification with simultaneous implantation of an iStent in the low nasal quadrant. The study was registered at ClinicalTrials.gov under the number NCT03807869.

### 2.1. Preoperative Examination

Detailed data was collected from all patients regarding prior treatment and surgery. All subjects underwent preoperative examination including measurement of IOP, BCVA, examination of the anterior and posterior segment of the eye, gonioscopy, and visual field examination (Humphrey, SITA Standard 30-2).

IOP was measured according to Advanced Glaucoma Intervention Study (AGIS) [16] using a Goldmann tonometer attached to a slit lamp and it was repeated twice. If the difference between two measurements was bigger than 3 mmHg, IOP was measured again. Average of two or three measurements was used to establish IOP and it was used for statistical analysis.

IOP measurements were taken at the same time of the day: between 8 and 10 am. The power of the artificial intraocular lens was calculated in all patients using the SRK/T regression formula on the IOL Master 700 (Carl Zeiss Meditec).

### 2.2. Surgical Technique

All surgeries were carried out at two centers (Department of Ophthalmology, Military Medical Institute in Warsaw and Clinic of Ophthalmology, Medical University of Białystok) by two surgeons (MR, JK). The specifications of iStent implantation technique and the iStent device have been described in detail before [17]. In brief, all surgeries were performed under local retrobulbar anesthesia with 2% Xylocaine. All patients underwent standard cataract phacoemulsification with implantation of an artificial posterior-chamber IOL into the capsular bag. A single first-generation iStent was inserted through the existing temporal incision in the corneal limbus into the nasal quadrant of Schlemm's canal, *ab interno*, using a Swan-Jacobs gonioscope. The injector was retracted after the stent's position was checked. The appearance of small blood reflux, of a self-limiting character, signified proper placement of the iStent. Removal of viscoelastic and sealing of the anterior chamber with a physiological salt solution, in order to obtain physiological pressure, concluded the operation. Eyes were treated postoperatively with topical containing steroids (Loteprednol) three times daily for four weeks, which then were tapered to BID after a week and antibiotic (Moxifloksacin) three times daily for two weeks, NSAIDS three times daily for 4 weeks, and followed up postoperatively through day 1, week 1, months 1, 3, 6, 12, and 24.

### 2.3. Postoperative Protocol

During the follow-up period, the anterior chamber and fundus were examined in addition to IOP and BCVA measurements. The postoperative period was evaluated taking into account complications and the number of antiglaucoma drugs.

IOP ≤ 6 mmHg was used as hypotension. Field of vision was performed at 6, 12, and 24 months after surgery. All antiglaucoma drugs were discontinued from the day of surgery. When the surgery did not bring the expected result, the medication was prescribed again in accordance with the EGS rules. The percentage of patients at the end of the observation period who were free of taking glaucoma drugs at IOP ≤ 18 mmHg or 20% of IOP reduction from a baseline was assumed as the surgical success.

### 2.4. Statistical Analysis

Statistical analysis was performed using the R program, version 3.5.1. The studied variables were presented using descriptive statistics. Nominal variables were compared between groups using the *χ*^2^ test or Fisher's exact test when the number of cells did not allow the use of the *χ*^2^ test. The normality of the distribution of quantitative variables was assessed using the Shapiro–Wilk test, skewness and kurtosis indicators, and visual assessment of histograms. Equality of variances was checked by Leven's test. Comparisons of tested groups of subjects were made using Student's *t*-test or Mann–Whitney U test, as appropriate. Comparative analysis of results between the beginning of the study and the 24th month was performed with the Wilcoxon test for dependent measurements. The significance level *α* = 0.05 was used, and all tests were two-sided.

Cumulative incidence of surgical success was calculated with Kaplan–Meier survival analysis method, including 95% confidence interval. Log-rank Cox–Mantel test was used to compare cumulative incidence of surgical success level between medicines subgroups. Additionally, Cox hazard ratio regression analysis model with IOP washout as covariate was used to identify factors significantly impacting surgical success.

## 3. Results

### 3.1. Demographics

This study involved 72 subjects, 52 females (72%) and 20 males (28%). Patients were divided into two groups based on the level of IOP after washout: 54 subjects had level of IOP < 26 mmHg before surgery and 18 had level of IOP ≥ 26 mmHg. The mean ± standard deviation age of all patients was 72.42 ± 9.17 years. From those, 62 patients (86%) had POAG, and 10 patients (14%) had PXG. The follow-up period was 20.33 ± 3.20 months in case of subjects with POAG and 19.33 ± 8.54 months in case of patients with PXG. There were not any statistically significant differences between the two groups in terms of demographics such as gender, age, type of glaucoma, or follow-up period. Demographics are summarized in Table 1.

### 3.2. Intraocular Pressure

For all subjects, the mean IOP before surgery was 22.05 ± 2.40 mmHg, and it was found that after 24 months it dropped by 6 mmHg (27%) to the average level of 16.20 ± 2.37 mmHg, *p* < 0.001, *MD* = −5.00, CI 95 [−6.50; −5.00] (Table 2, Supplementary Materials). For patients in IOP < 26 mmHg group before the surgery, average IOP was 21.03 ± 1.44 mmHg, and it dropped after 24 months by 5.0 mmHg (25.8%), *p* < 0.001, CI 95 [−6.00; −4.50] to the average level of 15.60 ± 2.12 mmHg. For the second group, where IOP ≥ 26, average IOP before the surgery was 26.00 ± 0.00 mmHg, and after 24 months of follow-up, it was lowered by 7.00 mmHg (28.6%), giving an average of 18.56 ± 1.81 mmHg, *p*=0.009, *MD*  = −7.00, CI 95 [−9.00; −6.00] (Table 3, Supplementary Materials). The difference in reduction of IOP between both groups was found statistically significant with *p*=0.003.

Difference between average IOP between both groups was found statistically significant for the first day (*p*=0.039) and then from the third day to the 24th month of the follow-up (*p* < 0.01). Every of these periods IOP was higher in the group of patients with IOP ≥ 26. Only in the 7th day and after the first month, postsurgical difference in IOP between both groups was not statistically significant (*p* > 0.05) (Table 4, Supplementary Materials).

**Figure 1:**
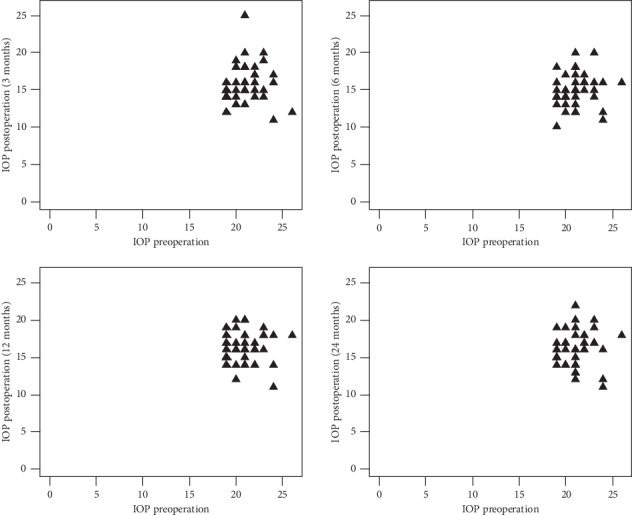
Scatterplot of IOP washout preoperation vs. IOP postoperation at 3, 6, 12, and 24 months after surgery.

In 12th month of study, 43.2%, CI 95 [28.3%; 58.9%] patients in IOP < 26 mmHg group had lower IOP ≥ 20% vs. baseline, while in IOP ≥ 26 mmHg group, it was 100%, CI 95 [73.5%; 100.0%], *p*=0.001. Difference in the number of patients with a drop in IOP ≥ 30% was also bigger for both groups for the 12th month: 15.9% patients in IOP < 26 group CI 95 [6.6%; 30.0%] vs. 66.7%, CI 95 [34.9%; 90.1%] in IOP ≥ 26 group and *p*=0.001. No statistically significant difference between reduced IOP values against baseline was found (Figure 1, Table 5, Supplementary Materials).

### 3.3. Medications

Patients were given average of 1.75 ± 0.89 medications. This mean number reduces at 24 months after surgery to 0.50 ± 0.90, *p* < 0.001, *MD* = −1.50, CI 95 [−1.50; −1.00] (Table 6, Supplementary Materials).

During the 24-month follow-up period, we found statistically significant differences between the groups in the number of drugs taken for each observation point with higher number of drugs in the IOP group ≥26 than in the IOP group <26 (*p* < 0.001 for the 6th and the 12th months of observation,*p*=0.026 for the 24th month) (Table 7, Supplementary Materials).

Relationship between the initial amount of antiglaucoma drugs in patients before the washout period was also analyzed (in groups of patients taking 0-1 drugs, 2 drugs, and 3–5 drugs) and their number after surgery at individual time points. During the 24-month observation period, statistically significant differences between the groups for the 6th month (*p*=0.014) and the 12th month of observation (*p*=0.028) were confirmed. In the 6th month of follow-up, patients taking 3–5 drugs had a significantly higher dose of drugs than patients from the 0-1 group of drugs, 0.91 ± 1.04 and 0.11 ± 0.4, respectively (*p*=0.006). Similarly, for the 12th month, patients receiving 3–5 drugs had a significantly higher dose of drugs than patients from the 0-1 group of drugs, 1.11 ± 1.17 versus 0.18 ± 0.50, respectively (*p*=0.011). At the end of the observation period, these values were 1.0 ± 1.58 versus 0.32 ± 0.57 (*p*=0.619) (Table 8, Supplementary Materials).

### 3.4. Surgical Success

**Figure 2:**
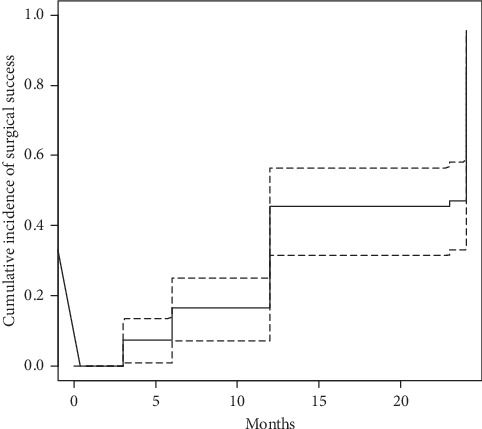
Kaplan–Meier survival curve for cumulative incidence of surgical success (dotted line is 95% confidence interval).

**Figure 3:**
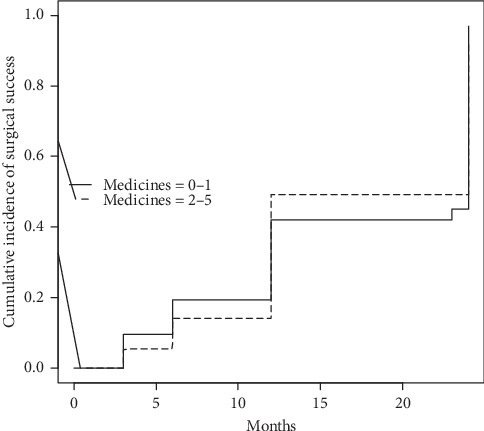
Kaplan–Meier survival curve for cumulative incidence of surgical success vs. the number of medicines.

At the end of the follow-up period, 71% of all patients did not take antiglaucoma medications and have the IOP ≤ 18 mmHg, 79% from the IOP < 26 mmHg group and 44% from the ≥26 mmHg group of patients (*p*=0.02) (Figures 2 and 3).

Cumulative incidence of surgical success was 45.5%, CI 95 [31.7%; 66.4%] at 12 months after surgery and 95.2%, CI 95 [85.5%; 98.4%] at 24 months after surgery.

Cumulative incidence of surgical success for group of patients receiving no or 1 medicine was 96.8%, CI 95 [87.8%; 99.5%] at 24 months after surgery and 93.6%, CI 95 [85.7%; 98.3%] in patients receiving 2–5 medicines at 24 months after surgery, difference not significant (log-rank *p*=0.900). Additional analysis using Cox hazard ratio model (with IOP washout level control) revealed no significant relationship between surgical success and age, sex, or number of medicines (Table 9, Supplementary Materials).

### 3.5. Best-Corrected Visual Acuity

There were no statistically significant differences in the level of visual acuity between the groups for any of the measurements during the 24-month observation period (Table 10, Supplementary Materials).

In the entire study group, preoperative visual acuity was 0.56 ± 0.24, and 24 months after surgery, it statistically significantly improved to 0.94 ± 0.13, *p* < 0.001, MD = 0.40, CI 95 [0.30; 0.45] (Table 11, Supplementary Materials). Only 2 of 72 patients (3%) had preoperative full visual acuity (level 1.0), and this number increased to 79% of patients 24 months after surgery (50 of 65).

### 3.6. Safety

All patients underwent cataract surgery followed by microbypass implantation into the Schlemm canal without intraoperative complications. In the postoperative period, the most common complication observed on the first days after surgery was a small amount of diffused blood in the anterior chamber and the folding of the Descemet membrane due to phacoemulsification. IOP increased in 7 patients (10%) not exceeding 25 mmHg and dropped back within a week after surgery. No cases of hypotension or other serious complications were observed, i.e., endophthalmitis, retinal detachment, or hemorrhage. During follow-up, 5 patients (5%) had worse BCVA compared to preoperative levels. In 2 cases, it was caused by the occurrence of a secondary cataract, and after the laser capsulotomy, the procedure returned to level 1.0. In the remaining 3 patients, it was caused by dry AMD and the choroidal membrane.

In two cases, bypass rotation was observed but without displacement into the anterior chamber. One case (from the IOP group> 26 mmHg) required reoperation due to nonnormalized IOP; it was excluded from the study and underwent trabeculectomy.

## 4. Discussion

The results of our study confirmed that iStent implantation is an effective method for lowering IOP in patients with POAG and PXG [10, 18]. The obtained decrease in IOP in the whole group of patients (27% versus baseline) is consistent with the studies of other authors [5, 6, 19, 20]. From the beginning of the observation period, postoperative IOP levels in the <26 mmHg group were statistically significantly lower compared to the ≥26 mmHg group and sustained like that at the end of the observation period (*p* < 0.05). The operation also allowed for a significant reduction of antiglaucoma drugs in the whole group of patients (from 1.75 ± 0.89 to 0.5 ± 0.90, *p* < 0.001, MD = −1.50 CI 95 [−1.50; −1.00]). Again, greater efficacy in this respect was obtained in the IOP group <26 mmHg than in the IOP ≥ 26 mmHg group at all points of the observation period reaching the point of 0.29 ± 0.52 vs. 1.33 ± 1.50 (*p*=0.026) at the end of follow-up. At the end of the follow-up period, 70% of all patients were free from antiglaucoma drugs: in the IOP <26 mmHg group as many as 79% subjects, in the IOP ≥ 26 group: 44% (*p* < 0.05), which is in the upper range of the results published before: 25.4%, 66%, and 74% [5, 7, 21]. All of the above parameters had a more favorable profile in the IOP group <26 mmHg compared to the group ≥26 mmHg.

Another issue is the effect of phacoemulsification alone on postsurgical decrease in IOP. According to various sources, it is on average: 1.4 mmHg, 1.9 mmHg, 1.55 mmHg, 1.88 mmHg, 2.9 mmHg, 3.1 mmHg, and 4.9–5.3 mmHg [22–26]. Depending on the type of glaucoma, the largest drop in IOP is in the eyes with closed-angle glaucoma and in PXG glaucoma (this effect is transient and after a year IOP gradually increases). Analyzing published studies, it can be concluded that the largest decrease in intraocular pressure occurs between the third and sixth months after surgery [27, 28]. From our previous studies, this effect remained at a similar level throughout the entire follow-up period which was 1 year, generally showing the lowest values after half a year of follow-up [29]. Hayashi et al. described an analogous IOP drop of 6.9 mmHg within 12 months after surgery, and even greater, as much as 7.2 mmHg, during 24 months after surgery. Generally, it can be concluded that this effect is most expressed during the first year after surgery [30], although there are reports in the literature that a reduction in IOP was noted even 10 years after surgery [31].

Analyzing the data from the above studies, it can also be seen that the decrease in IOP after surgery shows a strong inverse correlation with the preoperative depth of the anterior chamber, the width of the filtration angle, and the initial level of IOP.

These theories are confirmed in our study, where in the period of 3 and 6 months after surgery the number of patients with a decrease in IOP ≥ 20%, 30%, and 50% was statistically significantly higher in the group IOP ≥ 26 mmHg. At 12 months after surgery, these differences started to blur and were not statistically significant at the end of the observation period. Based on earlier work, we suspected that this is due to the cumulative effect of removing the lens and bypass implantation.

Studies conducted by Ferguson et al. also seem to confirm this thesis. Ferguson et al. found that a decrease in IOP after cataract surgery combined with iStent implantation was inversely proportional to the initial IOP. And so, the largest decrease in IOP was noted in the group with a pressure greater than 26 mmHg and it was even 11.28 mmHg compared to the group in the IOP 22–25 mmHg with a decrease by 7.69 mmHg and the IOP group of 18–21 mmHg where it was 3.48 mmHg [9]. However, as the authors themselves described in the study, it was rather a case series examined retrospectively, without specific criteria for inclusion and exclusion. Therefore, it is not known whether the authors took into account the configuration of the anterior segment of the eye and the anterior chamber depth before the procedure. After all, these are the factors that determine the degree of decline IOP after cataract surgery [29]. Based on this study, it can be concluded that the largest decrease in IOP is in the group with the highest IOP before surgery (which is also induced by cataract removal), but this is obtained through the largest amount of antiglaucoma medicines used. Thus, in Ferguson et al.'s study, the level of drugs before surgery in patients with IOP > 25 mmHg was 2.00, 24 months after surgery 1.33 (decrease by 34%), while in the group with IOP 22–24, the decrease was 67%. They achieved even smaller reduction in the number of drugs in the group with low IOP: from 1.35 to 0.90 (31%). It follows that the decrease in the amount of drugs after surgery is much smaller in the group with high IOP values.

Therefore, when considering the increase in resistance through the conventional outflow of fluid from the anterior chamber, not only the external wall of the Schlemm canal should be taken into account, but also the collector channel openings, which can play an important role here by changing the size of their entry surface depending on the IOP level. This thesis seems to be confirmed by studies using more than one microbypass during the procedure, which confirm that the decrease in IOP is not directly proportional to the number of stents introduced into the Schlemm canal. A 26% decrease in postsurgical IOP was observed after the implantation of only 1 stent combined with cataract phacoemulsification [21], 16–18% decrease after implantation of two bypasses with phacoemulsification [19, 32] and 20% decrease in IOP despite the implantation of 3 implants. Belovay et al. compared the use of 1, 2, and 3 stents simultaneously, and IOP reduction was obtained by 31%, 41%, and 41%, respectively. In theory, overcoming the resistance to aqueous humor outflow path at the trabecular level should lower the IOP to the pressure that equals the pressure inside the epidural veins. In practice, it is not the case: a reduction of IOP of 5–7 mmHg is obtained (depending on the group). This discrepancy confirms the theory of unidentified resistances existing outside the trabecular meshwork, which are more significant in patients with glaucoma than in healthy population [33, 34].

In another study, Seibolda et al. [35] implanted microbypass iStent in a group with IOP with low initial preoperative IOP (mean 14 ± 3.2 mmHg, range from 10 mmHg to 25 mmHg). Surgical success was defined in the criterion of a decrease in IOP from baseline by ≥ 20% or reduction of antiglaucoma drugs by at least one, and it was achieved in 76.1% of cases 12 months after surgery. The absolute decrease in IOP percentage was smaller than in other studies and amounted to 9.7% at the end of the observation period, which may indicate that the use of microbypass in pressures at a low level of a dozen or so mmHg also has limited hypotensive efficacy, although it allows most patients to discontinue antiglaucoma medication. This is confirmed by the research of Grieshber et al. about the correct autoregulation of aqueous humor flow in the IOP range between 10 and 20 mmHg [15]. The reduction of IOP with the use of iStent is limited by the level of IOP in the episcleral veins and does not allow obtaining a single-digit value without the additional use of antiglaucoma medicines.

Huan et al. raised an issue of uneven distribution of collector channels around the Schlemm canal. In their study, angiography of the Schlemm canal (live imaging of the outflow of aqueous humor from the Schlemm canal to collector channels in real time) was used to show segmental distribution of collector channels which also varies among the patients. This study suggests that the hypotensive success of iStent may be individual and depends on this distribution [36, 37].

Perhaps optimizing the site of microbypass implantation by choosing the place with the biggest number of collector channels is the future solution. In our case, iStents were implanted in the nasal quadrants because statistically the most collector channels are located there [38].

Our study is not without limitations; the major one is a homogenous group of patients (Caucasian race). We think that our findings would have been different in black subjects, as we know the resistance at the trabecular level is even greater. In addition, control groups come from two different centers, which may result in a slightly different course of research. However, before starting the study, the protocol was strictly defined, including inclusion and exclusion criteria. The strengths of our study include the long observation period and the washout period prior to the study.

## 5. Conclusion

An important inclusion criterion for the implantation of the iStent, in addition to the glaucoma stage, may be the assessment of permeability of distal outflow pathways, which include the Schlemm canal, water collectors, and episcleral veins, because the probability of success is higher then. The conclusions of the presented study may have practical value, as they explain why the effectiveness of the iStent microbypass implantation may be limited in eyes with chronic high IOP. The greatest reduction of IOP is achieved at high preoperative IOP levels, but maintaining target IOP requires reinclusion of antiglaucoma medicines. Similarly, in the group with low baseline IOP levels, the IOP reduction and the number of antiglaucoma drugs are the lowest. Most likely, the group that will benefit most from the microstent treatment is the one with low twenties, where both IOP levels and drug reduction are optimal. In cases with baseline IOP above 26 mmHg with intolerance of antiglaucoma medication, it may be appropriate to choose another type of surgical procedure. Perhaps, defining precisely, the target group of patients would result in even better hypotensive effectiveness of treatments using iStent implants.

## Figures and Tables

**Table 1:** Patients' demographic data.

	IOP < 26	IOP ≥ 26	*p* ^*∗*^
*N*	54	18
Age (years)	73.53 ± 8.25	69.17 ± 11.09	0.081

*Gender, n (%)*			
Female	40 (74.1)	12 (66.7)	0.761
Male	14 (25.9)	6 (33.3)	

*Glaucoma type, n (%)*			
POAG	48 (88.9)	14 (77.8)	0.413
PXG	6 (11.1)	4 (22.2)	0.413
Follow-up (months)	19.33 ± 8.54	20.33 ± 3.20	0.096

^*∗*^Student's *t*-test, *χ*^2^ test, or Fisher's exact test; data are presented as mean ± SD unless otherwise indicated. POAG: primary open-angle glaucoma; PXG: normal tension glaucoma.

## Data Availability

Readers can access the data supporting the conclusions of the study upon an e-mail request from the corresponding author. The names and personal data of the participants cannot be released due to ethical aspects.

## Supplementary Materials

Supplementary Materials The comparison of detailed pre-and postsurgery patients results (Tables 2–11).
