# Supplementary material for: The Effectiveness of First-Generation iStent Microbypass Implantation Depends on Initial Intraocular Pressure: 24-Month Follow-Up—Prospective Clinical Trial
Source: J Ophthalmol. 2020 Jun 23;2020:8164703. doi: 10.1155/2020/8164703 (PMC7330650; doi:10.1155/2020/8164703)
Supplement: Supplementary Materials — The comparison of detailed pre-and postsurgery patients results (Tables 2–11). [file 8164703.f1.pdf]

Supplementary materials

Table 2: Intraocular pressure (IOP) before and 24 months after surgery

**IOP, mmHg**	***n***	**Mean (*SD*)**	**Median (Range)**	***MD* (95% *CI*)**	***p******
Washout	65	22.05±2.40	21.00 (19.00;26.00)	-5.00 (-6.50; -5.00)	<0.001
24 months	65	16.20±2.37	16.00 (11.00;22.00)		

* Wilcoxon signed rank test, *MD* – difference in medians calculated as 24 months minus washout with 95% confidence interval

Table 3: Intraocular pressure (IOP) before and 24 months after surgery within groups

**IOP, mmHg**	***n***	**Mean (*SD*)**	**Median (Range)**	***MD* (95% *CI*)**	***p******
IOP < 26					
Washout	47	21.03±1.44	21.00 (19.00;24.00)	-5.00 (-6.00; -4.50)	<0.001
24 months	47	15.60±2.12	16.00 (11.00;20.00)		
IOP ≥26					
Washout	18	26.00±0.00	26.00 (26.00;26.00)	-7.00 (-9.00; -6.00)	0.009
24 months	18	18.56±1.81	19.00 (16.00;22.00)		

* Wilcoxon signed rank test, *MD* – difference in medians calculated as 24 months minus washout with 95% confidence interval.

Table 4: Intraocular pressure (IOP) mean values, median values, standard deviations, and range between the groups at specific times after surgery

**IOP (mmHg)**	**IOP < 26**		**IOP ≥26**		***MD* (95% *CI*)**	***p******
	**Mean (*SD*)**	**Median (Range)**	**Mean (*SD*)**	**Median (Range)**		
0	17.97±2.55	18.00 (12.00;24.00)	21.72±4.74	21.15 (14.20;30.90)	-3.15 (-5.30;-1.00)	0.002
Washout	21.17±1.70	21.00 (16.00;24.00)	27.56±2.99	26.00 (26.00;35.00)	-5.00 (-7.00;-5.00)	<0.001
1st day	16.57±5.15	16.00 (10.00;33.00)	19.67±4.21	18.00 (14.00;26.00)	-2.00 (-7.00;-0.01)	0.039
7th day	17.34±2.91	16.00 (13.00;26.00)	19.33±7.12	17.00 (13.00;34.00)	-1.00 (-4.00;2.00)	0.883
1st month	17.03±3.73	16.00 (10.00;29.00)	19.44±5.90	19.00 (11.00;33.00)	-3.00 (-4.00;0.01)	0.064

IOP (mmHg)	IOP < 26		IOP ≥26		*MD* (95% *CI*)	*p**
	Mean (*SD*)	Median (Range)	Mean (*SD*)	Median (Range)		
3rd month	14.76±2.60	15.00 (9.00;25.00)	18.04±3.66	18.00 (12.00;24.00)	-3.00 (-5.00;-2.00)	0.001
6th month	14.41±2.01	14.00 (10.00;19.00)	17.19±3.58	16.50 (12.00;23.00)	-2.50 (-5.00;-1.00)	0.006
12th month	15.45±2.35	16.00 (10.00;20.00)	17.57±1.91	18.00 (14.00;20.00)	-2.00 (-2.00;-1.00)	0.004
24th month	15.60±2.12	16.00 (11.00;20.00)	18.56±1.81	19.00 (16.00;22.00)	-3.00 (-5.00;-1.00)	0.001

∗ Mann-Whitney U test, *MD* – difference in medians calculated as IOP< 26 group minus IOP ≥26 group with 95% confidence interval.

Table 5: Proportion of eyes with IOP reduction >50%, >40%, >30% and >20% by time points between groups.

IOP reduction vs. baseline (washout)	≥50%	≥40%	≥30%	≥20%
1 month				
IOP<26(*n*=54) (%, 95% CI)	2.9 (0.1; 14.9)	2.9 (0.1; 14.9)	17.1 (6.6; 33.7)	40.0 (23.9; 57.9)
IOP ≥ 26 (*n*=18) (%, 95% CI)	12.5 (0.3; 52.7)	12.5 (0.3; 52.7)	37.5 (8.5; 75.5)	75.0 (34.9; 96.8)
**p*	0.341	0.341	0.332	0.118
3 months				
IOP<26(*n*=51) (%, 95% CI)	3.9 (0.5; 13.5)	7.8 (2.2; 18.9)	23.5 (12.8; 37.5)	52.9 (38.5; 67.1)
IOP ≥ 26 (*n* = 17) (%, 95% CI)	14.3 (1.8; 42.8)	35.7 (12.8; 64.9)	57.1 (28.9; 82.3)	85.7 (57.2; 98.2)
**p*	0.200	0.018	0.023	0.033
6 months				
IOP<26(*n*=52) (%, 95% CI)	0.0 (0.0; 7.8)	8.9 (2.5; 21.2)	31.1 (18.2; 46.7)	57.8 (42.2; 72.3)
IOP≥ 26 (*n* = 18) (%, 95% CI)	15.4 (1.9; 45.4)	30.8 (9.1; 61.4)	69.2 (38;6; 91.0)	92.3 (64.0; 99.8)
**p*	0.047	0.066	0.023	0.023

**IOP reduction vs. baseline (washout)**	**≥50%**	**≥40%**	**≥30%**	**≥20%**
12 months				
IOP<26(*n*=50)	0.0	2.3	15.9	43.2
(%, 95% CI)	(0.0; 8.0)	(0.1; 12.0)	(6.6; 30.0)	(28.3; 58.9)
IOP ≥ 26 (*n* = 17)	0.0	16.7	66.7	100.0
(%, 95% CI)	(0.0; 26.5)	(2.1; 48.4)	(34.9; 90.1)	(73.5; 100.0)
**p*	>0.999	0.113	0.001	0.001
24 months				
IOP<26(*n*=49)	0.0	2.9	25.7	51.4
(%, 95% CI)	(0.0; 10.0)	(0.1; 14.9)	(12.5; 43.2)	(34.0; 68.6)
IOP≥ 26 (*n* = 16)	0.0	0.0	50.0	87.5
(%, 95% CI)	(0.0; 36.9)	(0.0; 36.9)	(15.7; 84.3)	(47.3; 99.7)
**p*	>0.999	>0.999	0.217	0.111

Data presented as % of patients with IOP reduction of >50%, >40%, >30% and >20% vs. baseline with 95% confidence interval. IOP washout level was used as baseline level. * - χ^2 test or Fisher exact test.

Table 6: Amount of antiglaucoma medications before and 24 months after surgery

**Amount of Drugs**	***n***	**Mean (*SD*)**	**Median (Range)**	***MD* (95% *CI*)**	***p****
0	72	1.75±0.89	1.50 (1.00;4.00)	-1.50 (-1.50;-1.00)	<0.001
24 months	65	0.50±0.90	0.00 (0.00;4.00)		

* Wilcoxon signed rank test, *MD* – difference in medians calculated as 24 months minus washout with 95% confidence interval.

Table 7: Amount of antiglaucoma medications: mean values, median values, standard deviations, and range between the groups at specific times after surgery

**Amount antiglaucoma medications**	**IOP< 26**		**IOP ≥26**		***MD* (95% *CI*)**	***p***
	**Mean (*SD*)**	**Median (Range)**	**Mean (*SD*)**	**Median (Range)**		
0	1.35±0.65	1.00 (0.00;3.00)	2.89±1.18	3.00 (0.00;5.00)	-2.00 (-2.00;-1.00)	<0.001
6th month	0.16±0.43	0.00 (0.00;2.00)	1.25±1.00	2.00 (0.00;2.00)	-2.00 (-1.00;-0.01)	<0.001

12th month	0.22±0.48	0.00 (0.00;2.00)	1.38±1.04	2.00 (0.00;3.00)	-2.00 (-2.00;-0.01)	<0.001
24th month	0.29±0.52	0.00 (0.00;2.00)	1.33±1.50	1.00 (0.00;4.00)	-1.00 (-2.00;-0.01)	0.026

∗ Mann-Whitney U test, *MD* – difference in medians calculated as IOP washout < 26 group minus IOP washout ≥26 group with 95% confidence interval.

Table 8: Amount of antiglaucoma medications: mean values, median values, standard deviations, and range between the groups at specific times after surgery.

**Amount of Drugs**	**0-1 medicine**		**2 medicines**		**3-5 medicines**		***p***
	**Mean (*SD*)**	**Median (Range)**	**Mean (*SD*)**	**Median (Range)**	**Mean (*SD*)**	**Median (Range)**	
0	0.09 ±0.43	0.00 (0.00;2.00)	0.00 ±0.00	0.00 (0.00;0.00)	0.00 ±0.00	0.00 (0.00;0.00)	0.607
6th month	0.11 ±0.42	0.00 (0.00;2.00) [a]	0.32 ±0.58	0.00 (0.00;2.00)	0.91 ±1.04	0.00 (0.00;2.00) [a]	0.014
12th month	0.18 ±0.50	0.00 (0.00;2.00) [b]	0.31 ±0.48	0.00 (0.00;1.00)	1.11 ±1.17	1.00 (0.00;3.00) [b]	0.028
24th month	0.32 ±0.57	0.00 (0.00;2.00)	0.23 ±0.44	0.00 (0.00;1.00)	1.00 ±1.58	0.00 (0.00;4.00)	0.619

∗ Kruskal-Wallis test. *a-b*: significant differences in Dunn *post-hoc* test (a: $p = 0.006$, b: $p = 0.011$).

Table 9. Multivariate regression analysis with Cox hazard ratio method

Variable	Coefficient	SE (coefficient)	*p*	Exp (coefficient)
Age, years	-0.0004	0.02	0.981	0.99
Sex	0.18	0.40	0.654	1.19
Medicines (0-1 vs. 2-5)	-0.01	0.31	0.967	0.98

Table 10: Best corrected visual acuity (BCVA) mean values, median values, standard deviations, and range between the groups at specific times after surgery.

BCVA	IOP < 26 Mean (*SD*)	Median (Range)	IOP ≥26 Mean (*SD*)	Median (Range)	*MD* (95% *CI*)	*p**
0	0.51±0.24	0.50 (0.10;1.00)	0.54±0.27	0.60 (0.02;1.00)	-0.10 (-0.20; 0.10)	0.652
1st day	0.82±0.20	0.90 (0.30;1.00)	0.75±0.14	0.70 (0.60;1.00)	0.20 (-0.01; 0.20)	0.169
7th day	0.90±0.15	1.00 (0.50;1.00)	0.86±0.17	1.00 (0.60;1.00)	0.00 (-0.01; 0.20)	0.495
1st month	0.93±0.11	1.00 (0.63;1.00)	0.97±0.15	1.00 (0.70;1.25)	0.00 (-0.10; 0.01)	0.379
3rd month	0.88±0.20	1.00 (0.00;1.00)	0.89±0.22	1.00 (0.20;1.00)	0.00 (-0.01; 0.01)	0.636
6th month	0.90±0.18	1.00 (0.30;1.00)	0.92±0.16	1.00 (0.60;1.25)	0.00 (-0.01; 0.01)	0.937
12th month	0.93±0.15	1.00 (0.40;1.00)	0.93±0.13	1.00 (0.60;1.00)	0.00 (-0.01;0.01)	0.869
24th month	0.94±0.12	1.00 (0.60;1.00)	0.91±0.18	1.00 (0.50;1.00)	0.00 (-0.01; 0.01)	0.801

∗ Mann-Whitney U test, *MD* – difference in medians calculated as IOP washout < 26 group minus IOP washout ≥26 group with 95% confidence interval.

Table 11: Best corrected visual acuity before and 24 months after surgery.

VA	*n*	Mean (*SD*)	Median (Range)	*MD* (95% *CI*)	*p**
0	65	0.56±0.24	0.60 (0.10;1.00)	0.40 (0.30; 0.45)	<0.001
24 months	65	0.94±0.13	1.00 (0.50;1.00)		

* Wilcoxon signed rank test, *MD* – difference in medians calculated as 24 months minus washout with 95% confidence interval.
